# Modulating microtubule stability via α-tubulin acetylation partially restores Golgi fragmentation in spinal muscular atrophy

**DOI:** 10.55730/1300-0152.2798

**Published:** 2026-01-12

**Authors:** Pelin ZOBAROĞLU ÖZER, Memet GÖZÜBÖYÜK, Özge ÇETİN, Niko HENSEL, Güzin EMECEN, Adam MALIK, Melissa BOWERMAN, Peter CLAUS, Hayat ERDEM YURTER, Gamze BORA AKOĞLU

**Affiliations:** 1 Department of Medical Biology, Faculty of Medicine, Hacettepe University, Ankara, Turkiye; 2 Department of Medical Biology, Faculty of Medicine, Niğde Ömer Halisdemir University, Niğde, Turkiye; 3 Functional and Evolutionary Genetics Laboratory, Department of Biology, Faculty of Science, Hacettepe University, Ankara, Turkiye; 4 School of Medicine, Keele University, Staffordshire, United Kingdom; 5 Gene Transfer Technology, EGA Institute for Women’s Health, University College London, London, United Kingdom; 6 Department of Anatomy and Cell Biology, Faculty of Medicine, Martin Luther University of Halle-Wittenberg, Halle (Saale), Germany; 7 Wolfson Centre for Inherited Neuromuscular Disease, RJAH Orthopaedic Hospital, Oswestry, United Kingdom; 8 Center for Systems Neuroscience Hannover (ZSN), Hannover, Germany; 9 Laboratory for Molecular Neurosciences, Department of Psychiatry, Social Psychiatry, and Psychotherapy, Hannover Medical School, Hannover, Germany

**Keywords:** Spinal muscular atrophy, α-tubulin acetylation, HDAC6, Golgi fragmentation

## Abstract

**Background/aim:**

Spinal muscular atrophy (SMA) is a neurodegenerative disease caused by the loss of survival of motor neuron (SMN) protein. SMN deficiency leads to perturbations of the cytoskeleton, including microtubules, which are mainly involved in motility-related cellular processes. However, the molecular mechanisms of microtubule dysregulation in SMA remain elusive. Alpha (α)-tubulin is a structural component of microtubules, and its posttranslational modifications affect microtubule dynamics. Here, we aimed to investigate α-tubulin acetylation and related molecular mechanisms in SMA.

**Materials and methods:**

Two different SMA mouse models, the *Drosophila melanogaster* model and patient-derived fibroblasts, were used in the study. Western blot and quantitative microscopic analysis were performed to analyze α-tubulin acetylation and related mechanisms.

**Results:**

The acetylation level of α-tubulin was decreased in the *Drosophila* model and in SMA patient fibroblast cells but not in mouse models. This decrease in acetylation is associated with upregulation of the major tubulin deacetylase, HDAC6, in patient cells compared with healthy controls. Microtubules play a role in the organization of the Golgi apparatus, and we demonstrated that increasing α-tubulin acetylation by pharmacological inhibition of HDAC6 partially restored the fragmented morphology of the Golgi apparatus in SMA.

**Conclusion:**

Our findings provide new insight into the molecular basis of SMA, indicating that cellular pathologies, including abnormal Golgi morphology, are associated with microtubule dysregulations caused by altered α-tubulin posttranslational modifications and regulatory proteins. Our findings support that microtubule perturbations are part of SMA pathology.

## 1. Introduction

Spinal muscular atrophy (SMA) is a rare autosomal recessive neurodegenerative disease, primarily caused by deletions of the *survival of motor neuron 1* (*SMN1*) gene (Lefebvre et al., 1995). Progressive motor neuron degeneration in the anterior horn of the spinal cord and the brainstem, and muscle atrophy characterize SMA. Historically, SMA is classified into five groups, from severe to mild, based on achieved motor function and age of disease onset (Pearn, 1980; Wirth, 2021). Phenotypic differences between patients are commonly attributed to the modifier effect of a homologous gene, *SMN2*, since its copy number correlates with milder disease symptoms. However, due to the incorrect splicing of SMN2 pre-mRNAs, it could not compensate for the loss of full-length and functional SMN1 protein in patients (Wirth, 2021). The SMN is a ubiquitously expressed protein, localizing both to the cytoplasm and nucleus, where it interacts with several proteins to function in a wide range of biological processes, including snRNP biogenesis, splicing, and endocytosis (Singh et al., 2017; Chaytow et al., 2018; Mercuri et al., 2022). Studies with SMN-deficient models suggest that SMN is required for the homeostasis of the cytoskeleton (Cifuentes-Díaz et al., 2002; Rossoll et al., 2003; Bowerman et al., 2007; Oprea et al., 2008; Wen et al., 2010; Torres-Benito et al., 2011; Nölle et al., 2011; Miller et al., 2015; Hensel and Claus, 2017; Bora et al., 2019; Villalón et al., 2019; Bora et al., 2020; Siranosian et al., 2020; Özer et al., 2022). Perturbations of actin dynamics, caused by either impaired expression, localization, or posttranslational modifications (PTMs) of SMN-related proteins, including beta-actin, plastin 3, profilin IIa, and cofilin, have been demonstrated in SMA (Rossoll et al., 2003; Bowerman et al., 2007; Oprea et al., 2008; Nölle et al., 2011; Siranosian et al., 2020). In addition, accumulation of neurofilaments has been reported in SMA models and patients (Cifuentes-Díaz et al., 2002).

SMN deficiency also causes dysregulations in microtubule architecture and microtubule-associated proteins (Wen et al., 2010; Torres-Benito et al., 2011; Miller et al., 2015; Bora et al., 2019; Villalón et al., 2019; Bora et al., 2020; Özer et al., 2022). Microtubules play a role in motility-related cellular processes such as cell division, intracellular transport, and organelle positioning. Alpha (α) and beta (β) tubulin proteins form microtubule structures that originate from microtubule-organizing centers (MTOCs) such as the centrosome and/or Golgi apparatus (Conde and Cáceres, 2009; Wu and Akhmanova, 2017; Akhmanova and Kapitein, 2022). The Golgi attaches microtubule minus ends and serves as a platform for microtubule formation. In addition, microtubules and microtubule-associated proteins are involved in the formation of the Golgi. Depolymerization of microtubules by the chemical agent nocodazole fragments the Golgi, indicating an interconnection between these cellular structures (Minin, 1997). In SMA, Golgi fragmentation has been previously demonstrated; however, whether microtubule dysregulations contribute to this impairment is unknown (Custer et al., 2019).

Tubulin undergoes several chemically distinct PTMs, including acetylation, detyrosination, phosphorylation, polyglycylation, and polyglutamylation. These PTMs alter the structural properties of microtubules and their interactions with associated proteins (Janke and Magiera, 2020; Janke and Bulinski, 2011). Several SMA models displayed dysregulations of α-tubulin PTMs and microtubule-associated proteins, including MAP1B, MAP2, EB3, stathmin, and Tau (Wen et al., 2010; Torres-Benito et al., 2011; Miller et al., 2015; Bora et al., 2019; Villalón et al., 2019; Bora et al., 2020; Özer et al., 2022). We previously demonstrated that increased levels of MAP1B, together with tubulin tyrosine ligase (TTL), lead to the loss of α-tubulin detyrosination and microtubule stability (Bora et al., 2020). α-tubulin acetylation and its organization have also been reported to be impaired in SMA mice (Wen et al., 2010; Torres-Benito et al., 2011). Acetylation of lysine residue 40 (K40) of α-tubulin is a unique tubulin PTM that occurs on the lumenal side of the microtubule, and it is associated with stable microtubules due to the accumulation of long-lived microtubules. Acetylation is a reversible modification catalyzed by α-tubulin-N-acetyltransferase 1 (aTAT1), and deacetylation is primarily catalyzed by the major tubulin deacetylase, histone deacetylase 6 (HDAC6) (Janke and Montagnac, 2017; Hubbert et al., 2002).

Here, we analyzed α-tubulin acetylation in mouse and fly SMA models and in patient-derived fibroblasts. We found that α-tubulin acetylation was reduced in the *Drosophila* SMA model and in patient-derived fibroblast cells; however, we could not detect any change in mouse models. In patient cells, this reduction is associated with HDAC6, as pharmacological HDAC6 inhibition increased α-tubulin acetylation and restored the abnormally fragmented Golgi morphology. Our results suggest a new mechanistic model in which HDAC6 acts upstream of microtubule dysregulation and Golgi homeostasis in SMA. Together, our results highlight the importance of microtubules and their regulatory proteins in SMA pathophysiology.

## 2. Materials and methods

### 2.1. Cell culture studies

Primary fibroblast cells were obtained from the Coriell Cell Repository (with GM prefix) and the American Type Culture Collection (ATCC, with PCS prefix). Cells with an ML prefix have already been described (Nölle et al., 2011). Four SMA type I (GM09677, ML39, ML16 and ML17) and one type II (GM03813) patient cells, as well as cells of three healthy controls (GM08333, PCS-201-012 and ML35) were grown in high glucose Dulbecco’s Modified Eagle Medium (DMEM, Biological Industries or Gibco), containing 5% (v/v) fetal bovine serum (FBS), 1% penicillin/streptomycin at 37 °C, and 5% CO_2_ in an humidified incubator, as previously described (Dayangaç-Erden et al., 2009; Nölle et al., 2011). *SMN2* copy numbers of the cells are three for GM09677, GM03813, and ML16; and two for ML17 and ML39. Cells were either fixed for immunofluorescence analysis or were harvested into Radio-Immunoprecipitation Assay (RIPA) buffer (137 mM NaCl, 2 mM ethylenediaminetetraacetic acid (EDTA), 20 mM Tris-HCl pH 7, 1 mM sodium orthovanadate, 525 mM b-glycerophosphate, 1% (v/v) Triton-X-100, 1% (w/v) sodium deoxycholate), containing phosphatase and protease inhibitor cocktail tablets (Roche) for Western blot studies.

### 2.2. Immunofluorescence analysis

To visualize acetylated microtubules, fibroblast cells were fixed with 4% paraformaldehyde (PFA) for 10 min, then blocked with 1X phosphate-buffered saline (PBS) containing 1% bovine serum albumin (BSA) and 0.03% Triton X-100 for 1 h at room temperature. Cells were incubated with an antiacetyl α-tubulin (1:100, Sigma, Research Resource Identifier [RRID]: AB_609894) primary antibody at 4 °C overnight. Subsequently, an antimouse (1:500, Alexa Fluor 488, Invitrogen, RRID: AB_2534088) secondary antibody was added for 1 h at room temperature. Cells were mounted with 4′,6-diamidino-2-phenylindole (DAPI)-included Prolong Gold Antifade Solution (Invitrogen). They were then visualized using an upright fluorescence microscope (Carl Zeiss Axioplan 2) at 100× magnification (apochromatic objective, numeric aperture: 1.4). Fluorescence images were acquired with the same exposure settings and a short exposure time to avoid signal saturation. ImageJ software was used for fluorescence intensity analysis (National Institutes of Health, Bethesda, Maryland, USA). Briefly, using standard ImageJ settings (rolling ball radius: 50 pixels), background subtraction was applied to the images. The diameter of the nucleus and its center were automatically determined via the centroid function. Afterwards, to ensure that the analysis was performed in the same region across all cells in an unbiased manner, a 50 μm × 50 μm square was drawn around that center to measure fluorescence intensity in this defined perinuclear area, where signal differences were observed. Additionally, the cell surroundings were determined with a freehand tool to evaluate the fluorescence intensity of acetylated α-tubulin in whole cells. Images of 10 cells were analyzed in a blinded manner for each condition in two biological replicates.

### 2.3. Microtubule regrowth assay and morphological analysis of the Golgi apparatus

SMA (GM03813 and GM09677) and control fibroblast cells (GM08333 and PCS-201-012) were treated with 20 μM nocodazole (M1404, Sigma) or dimethyl sulfoxide (DMSO, Applichem) as a control for 2h to disrupt Golgi morphology by depolymerizing microtubules. Subsequently, cells were washed with 1X PBS five times to remove nocodazole. The microtubules were then repolymerized in a fresh culture medium containing tubastatin A (S8049, Selleckchem), a specific HDAC6 inhibitor, which was present throughout the microtubule regrowth period, at 4 μM for 4 h (Çetin et al., 2022). At the end of incubation, cells were fixed and costained with the following primary antibodies: antiacetyl α-tubulin (1:100, Sigma, RRID: AB_609894) and anti-GM130 (1:1000, Sigma, RRID: AB_532244) at 4 °C overnight. Antimouse (1:500, Alexa Fluor 488, Invitrogen, RRID: AB_2534088) and antirabbit (1:500, Alexa Fluor 568, Invitrogen, RRID: AB_10563566) secondary antibodies were applied as previously indicated. After mounting, cells were visualized at higher magnification (63×) with a fluorescence microscope (Carl Zeiss Axioplan 2), and images were analyzed for the area of Golgi as well as its colocalization with acetylated α-tubulin using ImageJ. Max entropy thresholding was applied to the images (15 images in three biological replicates, totaling >50 cells/condition), and the wand (tracing) tool in ImageJ was used to determine and quantify Golgi area. For colocalization analysis, 15 cells in three biological replicates (five cells/replicate) were analyzed in a blinded manner. The “Colocalization Threshold” plugin in ImageJ was used to determine Pearson’s correlation coefficients (r).

### 2.4. Mouse experiments

α-tubulin acetylation was analyzed in two different SMA mouse models. The Taiwanese mouse model is a severe SMA model with a murine *Smn* gene deficiency, but transgenic for the human *SMN2* gene, resulting in litters of SMA mice and heterozygous control mice (SMN^−/−^; *SMN2*^tg/0^ and SMN^+/−^; *SMN2*^tg/0^) (Hsieh-Li et al., 2000). Breeding and animal experiments were carried out in accordance with the German animal welfare law. Permission for breeding and animal experiments was approved by the Lower Saxony State Office for Consumer Protection and Food Safety (LAVES) (file no. 33.12-42502-04-15/1774). Mice were initially obtained from the Jackson Laboratory [stock number 005058 for the Taiwanese mouse model FVB.Cg-*Smn1*^tm1Hung^Tg (SMN2)2Hung/J]. SMA (P8) and control mice were decapitated, and then the thoracic T3–T13 segments of the spinal cord were dissected and frozen in liquid nitrogen immediately. The *Smn^2B/^*^−^ SMA mouse model was generated by breeding *Smn^2B/2B^* mice [generously provided by Dr. Rashmi Kothary (University of Ottawa), Dr. Lyndsay Murray (University of Edinburgh), and Professor Matthew Wood (University of Oxford) before being sent to Charles Rover for rederivation] with *Smn^+/^*^−^ mice (B6.Cg-*Smn1/J*, stock #007963, Jackson Laos) (Bowerman et al., 2012). The entire spinal cords of SMA (P18) and control mice were collected for protein extraction. All live procedures on wild type (WT) (C57BL/6 background) and *Smn^2B/^*^−^ SMA mice were performed in the Keele University Biological Sciences Unit, in accordance with the UK Home Office authorization [Animals Scientific Procedures Act (1986), UK Home Office Project Licence P99AB3B95].

### 2.5. *D. melanogaster* experiments

For *D. melanogaster* experiments, the *smn* gene mutant *smn^f05960^* (Dmel\PBac{WH}Smn/f05960), the ortholog of the *SMN1* gene, and its background line *w1118* (BL6326) (as WT) were used (Rajendra et al., 2007; Larkin et al., 2021). Both lines were obtained from the Indiana University Bloomington Drosophila Stock Center (BDSC). In the *smn^f05960^* line (BDSC stock number 18923), the *smn* gene harbors a piggyBac transposon insertion in the coding region (Rajendra et al., 2007). *Drosophila* lines were cultured in standard medium on a 12:12 light:dark cycle in an acclimatization chamber with 65% relative humidity (Markow and O’Grady, 2006). Flies were separated by age (0, 5, 10, 30, and 40 days old), placed in tubes (10 flies/tube), and frozen for 5 min in liquid nitrogen. Frozen flies were stored at −80 °C until protein extraction.

### 2.6. Western blot studies

Protein extractions from cells and mouse spinal cord tissues were performed in RIPA buffer using either sonication (Sonics) or homogenization (Fisher Scientific or Qiagen). To extract protein from flies, a liquid nitrogen-chilled ceramic mortar and pestle was used to disrupt whole flies, and then samples were immediately collected into sodium dodecyl sulfate (SDS)-containing lysis buffer [2 mM EDTA, 5M Tris-HCI pH 6.8, 20 % SDS (w/v), protease inhibitor cocktail tablet] and were boiled at 100 °C for 5 min before sonication on ice. Protein concentrations of samples were measured using the BCA assay (Pierce). Equal amounts of proteins (30–35 μg) were loaded into either 10% Mini-Protean TGX Stain-Free Protein gel (Bio-Rad) or 12% SDS-polyacrylamide gels after denaturation in a Laemmli buffer [80 mM Tris-HCl pH 6.8, 5% (v/v) 2-mercaptoethanol, 2% SDS (w/v), 0.01% (v/v) bromophenol blue]. A semidry or wet transfer system (Bio-Rad) was used to transfer protein samples onto nitrocellulose membranes. The following primary antibodies were used for blotting: mouse antiacetyl α-tubulin (1:1000, Sigma, RRID: AB_609894), rabbit anti-HDAC6 (1:1000, Cell Signaling, RRID: AB_10891804), mouse anti-SMN (1:1000, BD Transduction Laboratories, RRID: AB_397973), and mouse anti-α-tubulin (1:1000, Sigma, RRID: AB_477582) for the normalization. Horseradish-peroxidase-conjugated antimouse and antirabbit (Sigma, 1:8000, Amersham, 1:4000, respectively) as well as Alexa Fluor 546 conjugated antimouse (Sigma, 1:5000, RRID: AB_2737024) were used as secondary antibodies. Either stain-free imaging (ChemiDoc, Bio-Rad) or Supersignal West Femto (Pierce) was used to visualize protein bands with a CCD camera (GeneGnome, SynGene, and Intas). Densitometric analysis was performed using ImageJ or ImageLab software (Bio-Rad).

### 2.7. Statistics

Statistical analysis was performed using GraphPad Prism (version 8.02 for OS X, GraphPad Software, La Jolla, California, USA)^1^. The statistical tests used to evaluate significance were provided in the figure legends. Data with p < 0.05 were considered statistically significant. Data were presented as the mean with the standard error of the mean (SEM).

## 3. Results

### 3.1. α-tubulin acetylation levels are decreased in a *D. melanogaster* model of SMA

**Figure 1:**
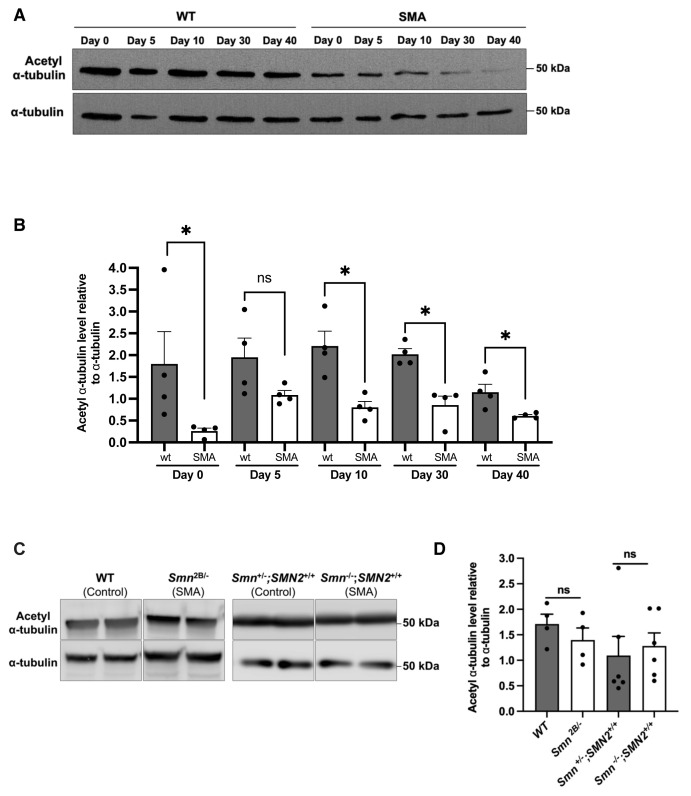
Level of α-tubulin acetylation in SMA model organisms. Representative Western blots and densitometric quantifications of α-tubulin acetylation in *D. melanogaster* (A, B) and mouse models (C, D) of SMA. For the *D. melanogaster* model, SMA (*smn^f05960^*) and WT control flies were collected on the day they emerged from their pupae (Day 0), and on the subsequent days (Days 5, 10, 30, and 40), n=4 (10 flies were pooled for each replicate). For mouse models of SMA, spinal cord tissues of *Smn^2B^*^/−^ (whole tissue) and *Smn*^−^*^/^*^−^*; SMN2^+/+^* (Taiwanese, T3–T13 segments) at symptomatic stages (P18 for *Smn^2B^*^/−^, P8 for *Smn*^−^*^/^*^−^*; SMN2^+/+^*) were used to quantify α-tubulin acetylation level and compared with controls. n=4 for *Smn^2B^*^/−^ mice, n=6 for *Smn*^−^*^/^*^−^*;SMN2^+/+^*. All normalizations were performed against total α-tubulin. Bars show the mean with the standard error of the mean (SEM). Statistical significance was tested by using the Mann–Whitney U test (*p < 0.05, ns: not significant).

Acetylation of α-tubulin has been associated with long-lived stable microtubule structures, and the loss of acetylation on lysine 40 affects microtubule-dependent cellular functions (Cambray-Deakin and Burgoyne, 1987; Janke and Bulinski, 2011; Aguilar et al., 2014; Janke and Montagnac, 2017; Portran et al., 2017). To evaluate α-tubulin acetylation in SMA, we employed an in vivo *D. melanogaster* model with a hypomorphic *smn^f05960^* allele (Sen et al., 2011). In this model, as previously described, prolonged developmental time and reduced locomotor behavior were observed compared with controls (Praveen et al., 2014; Spring et al., 2019; Grice and Liu, 2022). Flies were collected on different days of their lifespan for protein extraction. Western blots showed that acetylated α-tubulin level was significantly downregulated in SMA flies compared with controls (Figures 1A and 1B). To confirm this in a mammalian system and in central nervous system (CNS) tissue, we employed SMA mouse models to investigate α-tubulin acetylation. We analyzed spinal cord tissue from two different SMA mouse models, severe Taiwanese mice (T3–T13 segments) and less severe *Smn^2B^*^/−^ mice (whole tissue), having different genetic backgrounds (Hsieh-Li et al., 2000; Bowerman et al., 2012). However, unlike in *Drosophila*, no significant change in α-tubulin acetylation levels was observed in the spinal cord tissues of either *Smn^2B^*^/−^or Taiwanese mouse models at symptomatic stages, possibly due to dilution effects by neighboring nonaffected cells, which may be more prominent in number (Figures 1C and 1D). Together, these findings may arise from either differential regulation of α-tubulin acetylation or from tissue- or context-dependent mechanisms in different model organisms.

### 3.2. Reduced α-tubulin acetylation in SMA patient fibroblasts is associated with increased levels of HDAC6

**Figure 2:**
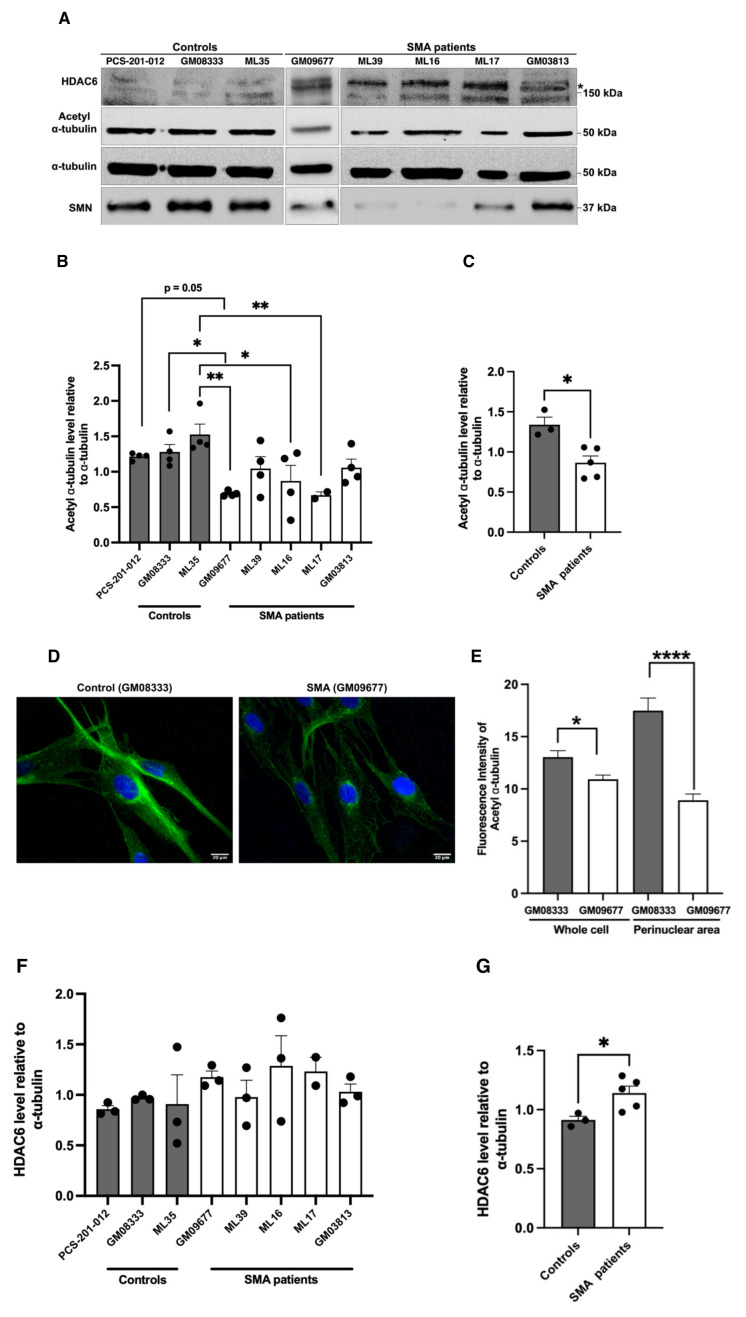
Acetylated α-tubulin and HDAC6 levels in SMA patient-derived fibroblast cells. Representative blots and densitometric quantifications of acetylated α-tubulin levels in SMA fibroblast cells compared with healthy control cells (A, B). Overall acetylated α-tubulin levels shown in SMA and control fibroblast cells (C). Representative immunofluorescence images of control (GM08333) and SMA type I (GM09677) fibroblast cells stained by acetylated α-tubulin antibody (D). Fluorescence intensity analysis of acetylated α-tubulin in whole cells as well as in the perinuclear area, which was determined using the center of the nucleus as a reference point. A 50 μm × 50 μm square was drawn around the center as a region of interest and analyzed in all cells (E). Images of 10 cells were analyzed in a blinded manner in independent culture experiments (n=2). Scale bar, 20 μm. Densitometric analysis of HDAC6 level in SMA patient and control fibroblast cells relative to total α-tubulin (A, F). Overall HDAC6 levels shown in SMA and control fibroblast cells (G). Patient fibroblast cells shown in the blots and bar graphs are SMA Type I: GM09677, ML39, ML16, and ML17, and Type II: GM03813. Control fibroblast cells are PCS-201-012, GM08333, and ML35. The molecular weights of the marker are shown on the right side of the blots. In some cells, HDAC6 appears as multiple bands around 150 kDa, which might reflect splice variants or posttranslationally modified products. The asterisk indicates the HDAC6 product at approximately 160 kDa. Results were presented as bar graphs, with SEM and replicates shown. ML17 was analyzed twice due to sample limitations. One-way ANOVA with Dunnett’s multiple comparison (B, F) or Mann-Whitney U (C, E, G) tests were used to test statistical significance. *p < 0.05, **p < 0.01 ****p < 0.0001.

Loss of α-tubulin acetylation has not been previously reported in SMA patients. Therefore, we analyzed α-tubulin acetylation in fibroblasts from five SMA patients (four Type I and one Type II) and three healthy controls. Western blot results demonstrated a significant reduction in acetylated α-tubulin level in SMA fibroblasts compared with healthy controls (Figures 2A–2C). Immunofluorescence analyses demonstrated the prominent loss of acetylated microtubule networks, especially in the perinuclear area of SMA fibroblast cells (Figures 2D and 2E). To mechanistically address reduced α-tubulin acetylation, we analyzed the level of the major α-tubulin deacetylase, HDAC6, and detected a moderate, but statistically significant, overall upregulation in SMA fibroblast cells (Figures 2A, 2F, and 2G). These findings indicate that altered HDAC6 activity contributes to reduced α-tubulin acetylation in SMA patient cells.

### 3.3. HDAC6 inhibition partially restored Golgi fragmentation in SMA fibroblast cells

**Figure 3:**
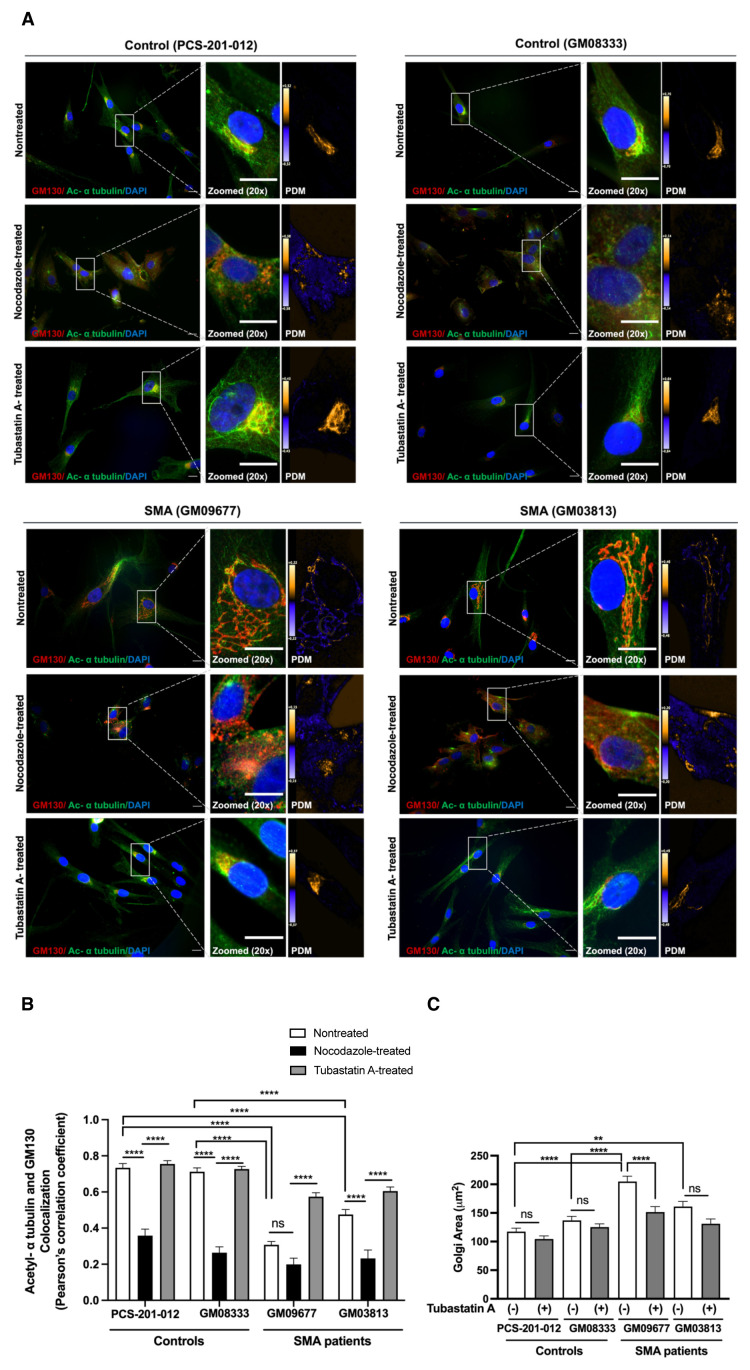
Relationship between Golgi fragmentation and α-tubulin acetylation in SMA patient-derived fibroblast cells. Immunofluorescence costaining of acetylated α-tubulin (green) and a cis-Golgi marker (GM130, red) in SMA and control fibroblast cells (A). Representative images of nontreated, nocodazole-treated, and tubastatin A-treated SMA and control cells were provided, along with merged images at 20× magnification. The product of the differences from the mean (PDM) images was also given to show the colocalization, with orange representing colocalized pixels and blue indicating segregation. Scale bar, 20 μm. Colocalization analysis of acetylated α-tubulin and Golgi in nontreated, nocodazole-treated, and tubastatin A-treated SMA and control fibroblast cells (B). Fifteen cells were analyzed in a blinded manner in three independent culture experiments, and Pearson’s correlation coefficient was calculated by ImageJ. Analysis of the Golgi area in nontreated and tubastatin A-treated SMA and control fibroblast cells (C). The Golgi area was measured as a readout of fragmentation, since intact Golgi occupies a smaller area compared with fragmented Golgi. A total of >50 cells were used for each condition in three independent culture experiments to analyze the Golgi area in a blinded manner. Two patients having different levels of SMN (Type I: GM09677 and Type II: GM03813) and two controls (PCS-201-012 and GM08333) were used for all analyses. Bars show mean with SEM. Two-way ANOVA with Tukey’s multiple comparison test was used to analyze statistical significance (B, C). ** p < 0.01, ****p < 0.0001, ns: not significant.

Microtubules are involved in the regulation of organelle morphology, including the Golgi, which has been shown to be fragmented in SMA patient-derived fibroblasts (Thyberg and Moskalewski, 1999; Barlan and Gelfand, 2017; Custer et al., 2019). Therefore, we evaluated whether the impaired Golgi morphology is associated with reduced α-tubulin acetylation in SMA. We performed microtubule regrowth experiments in two SMA patient fibroblast cell lines with different SMN levels (GM09677 and GM03813). First, we fragmented the Golgi by depolymerizing the microtubule network with nocodazole. Subsequently, nocodazole was washed out, allowing microtubules to repolymerize. In another condition, we used tubastatin A, a specific HDAC6 inhibitor, to pharmacologically increase α-tubulin acetylation. Coimmunostainings with a cis-Golgi marker, GM130, and acetylated α-tubulin antibodies were performed, and the colocalization of Golgi and acetylated α-tubulin, as well as the Golgi area, was used as a readout. Quantitative image analysis showed a significant reduction in the colocalization of Golgi and acetylated α-tubulin in nontreated SMA fibroblast cells, compared with nontreated healthy controls (Figures 3A and 3B). In addition, the Golgi area in SMA fibroblasts was significantly larger than in controls under nontreated conditions, due to Golgi fragmentation. Nocodazole treatment mostly reduced colocalization of the Golgi and acetylated α-tubulin, and tubastatin A partially restored it in SMA fibroblasts. Tubastatin A treatment also reduced the Golgi area in SMA fibroblasts, especially the cell line with the greatest SMN loss. These findings indicate that microtubule acetylation during polymerization protects Golgi integrity and that increasing acetylation by HDAC6 inhibition partially improves Golgi morphology in SMA patient fibroblast cells (Figures 3A and 3C).

## 4. Discussion

SMN deficiency causes a wide range of cellular pathologies, some of which can potentially be associated with altered microtubules. Microtubules have not been studied in detail in SMA, although previous findings showed dysregulations in microtubule regulatory proteins as well as their stability (Wen et al., 2010; Torres-Benito et al., 2011; Miller et al., 2015; Bora et al., 2019; Villalón et al., 2019; Bora et al., 2020; Özer et al., 2022). In this study, we demonstrated a reduced α-tubulin acetylation in SMA. α-tubulin proteins are acetylated after microtubule polymerization and accumulate in long-lived, stable microtubules (Janke and Bulinski, 2011; Janke and Magiera, 2020). Due to their postmitotic nature, neuronal microtubules are stable, a property that is crucial for maintaining cellular morphology and axonal transport (Baas et al., 2016). Loss of α-tubulin acetylation has been reported in different neurodegenerative disease models and is associated with defective axonal transport (Dompierre et al., 2007; D’Ydewalle et al., 2011; Cartelli et al., 2012; Godena et al., 2014; Guo et al., 2017). In SMA, reduced α-tubulin acetylation has been found in the sciatic nerve of SMA-like mice. On the other hand, analysis of central nervous system tissues in *Smn^2B/^*^−^ mice revealed no alteration in α-tubulin acetylation in the brain, whereas the reduction observed in the spinal cord appeared to be associated with total α-tubulin levels (Wen et al., 2010; Villalón et al., 2019). According to our findings, α-tubulin acetylation levels in the spinal cords of both *Smn^2B/^*^−^ and Taiwanese mice were not significantly different from their respective controls. Different findings obtained from mouse models may be attributable to differential regulation of microtubule acetylation between the peripheral and central nervous systems, as well as to cell-type-specific regulation within tissues. Intriguingly, we found a significant decrease in α-tubulin acetylation in the *Drosophila* SMA model at all time points except day 5. The lack of significance at day 5 may reflect age-related regulation of microtubule acetylation. Since a whole organism was used, these findings also suggest that α-tubulin acetylation may be decreased in peripheral non-CNS tissues. Consistent with this, we demonstrated a significant decrease in α-tubulin acetylation in SMA patient-derived fibroblast cells. A reduction in the acetylated microtubule network was prominent, especially in the perinuclear area containing the MTOC, in patient cells. There is an inverse relationship between α-tubulin acetylation and HDAC6; therefore, we analyzed HDAC6 levels in these cells (Hubbert et al., 2002). Collectively, HDAC6 levels were significantly upregulated in SMA patient cells compared with controls. Since HDAC6 is the major tubulin deacetylase, these results suggest that it could be responsible for the reduced acetylation of α-tubulin in the absence of SMN. Pharmacological inhibition of HDAC6 activity has been shown to increase acetylation of α-tubulin, leading to the restoration of several perturbations, including defective axonal transport, impaired mitochondria dynamics, and fragmented Golgi morphology (Li et al., 2012; Simões-Pires et al., 2013; Deakin and Turner, 2014; Guedes-Dias et al., 2015; Guo et al., 2017). Among all these abnormalities, we focused on the Golgi because of its bidirectional regulation with microtubules (Thyberg and Moskalewski, 1993; Custer et al., 2019). Golgi morphology depends on microtubule networks, while the Golgi serves as an MTOC in some cell types, including fibroblasts (Akhmanova and Kapitein, 2022). Acetylated microtubules are enriched at the Golgi, and they are required for the establishment and maintenance of the organelle (Thyberg and Moskalewski, 1993; Ide et al., 2021). Golgi fragmentation has been previously reported in SMA patient fibroblasts, and SMN overexpression has been shown to restore Golgi morphology (Custer et al., 2019). We also observed Golgi fragmentation in SMA patient cells and analyzed whether reduced α-tubulin acetylation affects the Golgi. We disrupted the Golgi structure by nocodazole-induced microtubule depolymerization and subsequently allowed the networks to regrow in the presence of an HDAC6 inhibitor, tubastatin A. We found that perturbations in acetylated microtubule-Golgi colocalization and Golgi area in SMA patient cells could be partially restored by tubastatin A treatment, suggesting that increasing α-tubulin acetylation during microtubule polymerization partially improves Golgi morphology in a disease-specific manner. These findings were more pronounced in SMA Type I fibroblasts than in Type II, supporting the notion that SMN levels are important for Golgi architecture. However, it remains unknown how fragmentation affects Golgi function and whether inhibiting HDAC6 activity can result in functional recovery. HDAC inhibition has been extensively studied in SMA to enhance SMN levels with pan-HDAC inhibitors. Recent reports demonstrated that combinational treatment of nusinersen-like antisense oligonucleotides with HDAC inhibitors enhances the correct splicing of *SMN2* and its expression (Hensel et al., 2020; Pagliarini et al., 2020; Marasco et al., 2022). Since pan-HDAC inhibitors also affect HDAC6 activity, it is plausible that microtubule acetylation and dynamics could also be affected (Osseni et al., 2020).

SMA is no longer considered a sole motor neuron disease, as abnormalities are now observed in other organs beyond the spinal cord and brainstem (Shababi et al., 2010; Somers et al., 2016; Allardyce et al., 2020; Yeo and Darras, 2020). The organization of microtubules is different among cell types (Sallee and Feldman, 2021). For example, in fibroblasts, microtubules are more dynamic than in neurons, as they are required for cell proliferation, adhesion, and migration (Ren et al., 1999; Akhmanova and Kapitein, 2022). Since α-tubulin acetylation marks stable microtubules, decreased level of acetylation in SMA patient fibroblasts implicated reduced microtubule stability, which is in accordance with our previous findings in a motor neuron-like SMA model (Bora et al., 2020).

**Figure 4:**
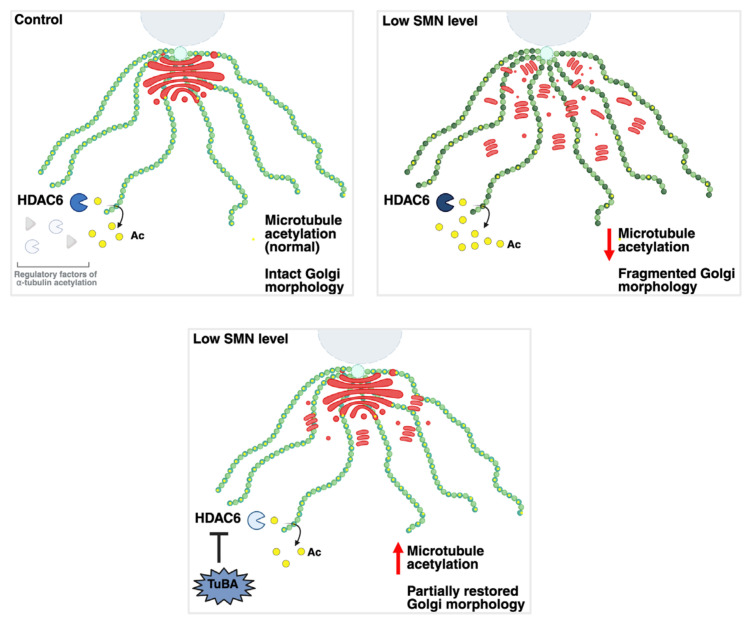
Proposed mechanistic model for reduced α-tubulin acetylation, Golgi fragmentation, and its partial morphological restoration in SMA. TuBA and Ac indicate tubastatin A and acetyl groups, respectively. Created in BioRender^2^.

Taken together, we propose a mechanistic model suggesting that increased HDAC6 activity leads to the reduction of acetylated microtubule network, which no longer supports Golgi integrity in conditions with low SMN levels. Increasing α-tubulin acetylation via HDAC6 inhibition during microtubule polymerization partially improves Golgi morphology (Figure 4).

Currently available SMN-enhancing therapies are changing the natural history of the disease; however, understanding all aspects of SMN-related molecular alterations, including microtubules, is still needed to develop combinatorial therapeutic approaches.

## Footnotes

1 GraphPad Software (2019). GraphPad Prism version 8.02 for macOS [online]. Website: https://www.graphpad.com/

2 Created in BioRender. Bora, G. (2026) https://BioRender.com/a61f176
